# Diosmin Inhibits Glioblastoma Growth through Inhibition of Autophagic Flux

**DOI:** 10.3390/ijms221910453

**Published:** 2021-09-28

**Authors:** Yung-Lung Chang, Yao-Feng Li, Chung-Hsing Chou, Li-Chun Huang, Yi-Ping Wu, Ying Kao, Chia-Kuang Tsai

**Affiliations:** 1 Department of Biochemistry, National Defense Medical Center, Taipei 11490, Taiwan; ylchang@mail.ndmctsgh.edu.tw (Y.-L.C.); emily7781@hotmail.com (L.-C.H.); nico539982@yahoo.com.tw (Y.-P.W.); 2 Department of Pathology, Tri-Service General Hospital, National Defense Medical Center, Taipei 11490, Taiwan; liyaofeng@ndmctsgh.edu.tw; 3 Department of Neurology, Tri-Service General Hospital, National Defense Medical Center, Taipei 11490, Taiwan; choutpe@yahoo.com.tw; 4 Graduate Institute of Medical Sciences, National Defense Medical Center, Taipei 11490, Taiwan; mtlittlcat@gmail.com; 5 Division of Neurosurgery, Department of Surgery, Taipei City Hospital Zhongxing Branch, Taipei 10341, Taiwan; 6 University of Taipei, Taipei 10608, Taiwan

**Keywords:** diosmin, glioblastoma multiforme, autophagy

## Abstract

Diosmin, a natural flavone glycoside acquired through dehydrogenation of the analogous flavanone glycoside hesperidin, is plentiful in many citrus fruits. Glioblastoma multiforme (GBM) is the most malignant primary brain tumor; the average survival time of GBM patients is less than 18 months after standard treatment. The present study demonstrated that diosmin, which is able to cross the blood–brain barrier, inhibited GBM cell growth in vitro and in vivo. Diosmin also impeded migration and invasion by GBM8401and LN229 GBM cells by suppressing epithelial-mesenchymal transition, as indicated by increased expression of E-cadherin and decreased expression of Snail and Twist. Diosmin also suppressed autophagic flux, as indicated by increased expression of LC3-II and p62, and induced cell cycle arrest at G1 phase. Importantly, diosmin did not exert serious cytotoxic effects toward control SVG-p12 astrocytes, though it did reduce astrocyte viability at high concentrations. These findings provide potentially helpful support to the development of new therapies for the treatment of GBM.

## 1. Introduction

Highly malignant glioblastoma multiforme (GBM) is the most common primary central nervous system tumor in adults [1,2]. The anticipated survival time of GBM patients after standard treatment, including maximum surgical resection and postoperative chemoradiotherapy, is less than 18 months [3,4]. Hence, much effort is being made to identify new treatments for GBM. In that regard, there is growing interest in flavonoids, in part because of their anti-inflammatory and antioxidant properties, but also because they exhibit potential antitumor activity [5,6]. Diosmin, a natural flavone glycoside acquired through dehydrogenation of the analogous flavanone glycoside hesperidin, is plentiful in many citrus fruits. Up to now, diosmin has been applied to vessel-protective treatments for hemorrhoids and varicose veins. In addition, Liu et al. reported that diosmin may exert protective effects in ischemic brain tissue [7]. Notably, diosmin also exhibits an ability to inhibit prostate cancer growth [8]. Moreover, diosmin crosses the blood–brain barrier, as evidenced by its detection in rat brain tissues with ultrahigh performance liquid chromatography-coupled masses (UHPLC-MS) analysis [9].

To assess diosmin’s potential as an anti-GBM drug, we evaluated GBM cell growth in the presence of various concentrations of diosmin. In addition, because aggressive invasion is a lethal characteristic of GBM, we investigated diosmin’s effect on cell migration and epithelial–mesenchymal-like transition (EMT-like) markers in GBM. Finally, we used gene microarray analysis to shed light on part of diosmin’s molecular mechanism.

## 2. Materials and Methods

### 2.1. Culture of GBM Lines

GBM8401 and LN229 human GBM cell lines were cultured in Dulbecco’s Modified Eagle Medium (DMEM) supplemented with 2% fetal bovine serum, penicillin, and streptomycin. All cells were maintained at 37 °C under 5% CO_2_ and 95% air, as previously described by Tsai et al. [10,11]. LN229 cells containing the luciferase gene for infrared luminance (iRL) imaging were kindly provided by Dr. Ying Chen. These cells were obtained through stable transfection of pLuc2-iRFP and selected using a FACSAria Fusion Sorter. LN229 (+iRL) cells were cultured in Roswell Park Memorial Institute (RPMI) 1640 medium.

### 2.2. Chemicals

The following chemicals were used: diosmin (Sigma, Burlington, MA, USA), torin (AdooQ BioScience, Irvine, CA, USA), and chloroquine (CQ) (AdooQ BioScience).

### 2.3. Colony Formation Assays

Cells (1000 cells per well) were seeded into 6-well soft agar plates and cultured for 2 weeks. The colonies formed were then stained with 0.5% crystal violet, after which ImageJ software (National Institutes of Health, Bethesda, MD, USA) was used to calculate the total area of colonies larger than 0.5 mm.

### 2.4. Western Blotting

For western blotting, cells were washed in PBS and then homogenized in RIPA buffer (100 mM Tris-HCl, 150 mM NaCl, 0.1% SDS, and 1% Triton-X-100) at 4 °C. Following separation of the proteins by electrophoresis and their transfer to PVDF membranes, the primary antibodies used were against cyclin D1 (Cell Signaling Technology, Danvers, MA, USA), cyclin B1 (Cell Signaling Technology, Danvers, MA, USA), CDK1 (Cell Signaling Technology, Danvers, MA, USA), E-cadherin (Cell Signaling Technology, Danvers, MA, USA), Snail (Cell Signaling Technology, Danvers, MA, USA), Twist (Santa Cruz Biotechnology, Dallas, TX, USA), p62 (Santa Cruz Biotechnology), LC3-II (Cell Signaling Technology, Danvers, MA, USA), p21 (Cell Signaling Technology, Danvers, MA, USA), CDK2 (Cell Signaling Technology, Danvers, MA, USA), vimentin (Cell Signaling Technology, Danvers, MA, USA), N-cadherin (Cell Signaling Technology, Danvers, MA, USA), CCL2 (Abcam Inc., Cambridge, MA, USA), and ACTN (Santa Cruz Biotechnology, Dallas, TX, USA). Protein bands were visualized using enhanced chemiluminescence (ECL) Detection Reagent (GE Healthcare, Piscataway, NJ, USA).

### 2.5. Migration and Invasion 

We used wound healing assays to evaluate cell migratory activity. LN229 and GBM8401 cells were seeded into 6-well plates and grown to confluence to establish a monolayer. We then scratched the monolayer with a P200 pipette tip to create a wound. After 16 h of treatment with the indicated concentrations of diosmin, the wound area was photographed and measured using ImageJ software (NIH, Bethesda, MD, USA).

We performed the transwell invasion assays by seeding 1.5 × 10^5^ GBM8401 or 2 × 10^5^ LN229 glioma cells in serum-free DMEM with or without diosmin into the upper chambers of transwell (BD Biosciences) plates in which the interchamber membranes were coated with a thin layer of Matrigel. The lower chambers contained DMEM with 2% FBS. After incubating the chambers at 37 °C in a CO_2_ incubator for 16 h, glioma cells that had migrated to the lower side of the membrane were fixed in 4% formaldehyde and stained with 0.1% crystal violet (Merck Millipore, Burlington, MA, USA). The numbers of migrated cells in three arbitrarily selected fields in each membrane were then counted under a microscope.

### 2.6. Flow Cytometric Analysis of Cell Viability and Proliferation, Cell Cycle Progression, Autophagy, and Senescence

The effect of diosmin on cell viability was determined using MTS assays. GBM8401 and LN229 cells were plated in a 96-well plate and incubated with various concentrations of diosmin for 24, 48, or 72 h, after which MTS solution (20 μL/well) was added, and the absorbance at 490 nm was recorded using a Varioskan™ LUX multimode microplate reader. For proliferation analysis, cells were stained using a FITC-5′-bromo-2′-deoxyuridine (BrdU) Flow Kit (BD Biosciences, San Jose, CA, USA) according to the manufacturer’s instructions. For cell cycle assays, fluorescence-activated cell sorting (BD Biosciences, San Jose, CA, USA) was used to characterize the DNA within the cells. To detect the presence of autophagy in diosmin-treated glioma cells, an Autophagy Detection Kit (Abcam) was used according to the manufacturer’s instructions. For senescence assays, cells were labeling with C_12_FDG that could produce a fluorescent product when cleaved by β-galactosidase inside the cell. The fluorescein signal of C_12_FDG was quantified on the FL1 detector [10].

### 2.7. Immunofluorescent Staining and Quantification

Cells were incubated for 48 h on glass coverslips in the presence of diosmin and then fixed for 5 min in 10% formaldehyde in PBS, permeabilized with 0.1% Triton X-100 in PBS, blocked with 1% BSA (Sigma, Burlington, MA, USA) in PBS, and incubated with rabbit anti-human LC3 (Cell Signaling Technology, Danvers, MA, USA) in 1% BSA overnight at 4 °C. Thereafter, the cells were washed three times with TBST and incubated with Alexa 488-goat anti-rabbit IgG secondary antibody (1:200; Jackson, West Grove, PA, USA) in 1% BSA for 1 h at room temperature. After three more washes with TBST for 5 min each, the nuclei were stained with DAPI (Sigma, Burlington, MA, USA) for 5 min at room temperature. Finally, the coverslips were mounted on glass slides with mounting medium (Thermo Fisher Scientific, Waltham, MA, USA) and examined under a Leica DM2500 microscope.

The LC3-II immunofluorescence was quantified using the intensity quantification function of ImageJ. After determining the proper threshold for positive immunoreactivity, we applied this same setting to all the immunofluorescent images. We then calculated the average stained area at each cell (pixel^2^/cell).

### 2.8. Glioma Orthotopic Xenograft in Nude Mice

All procedures were performed in accordance with the humane and customary care and use of experimental animals and followed a protocol approved by the Institutional Animal Care and Use Committee of the National Defense Medical Center. Female nude mice, approximately 5–6 weeks of age on arrival, were purchased from the National Laboratory Animal Center and kept in microisolator cages under pathogen-free conditions on a 12-h light/12-h dark schedule for a week. The orthotopic procedure was as previously described [10,11]. After intraperitoneal anesthetization, athymic nude mice were placed in a stereotactic frame, a midline cranial incision was made, and a 5-mm burr hole was made in the right side of the skull, 2.5 mm lateral to the midline and 0.4 mm posterior to the bregma. A total of 1 × 10^5^ LN229 (+iRL) glioma cells in 5 μL of culture medium and Matrigel (Corning, NY, USA) were intracranially implanted into a site 3 mm below the brain surface of each mouse. Mice exhibiting similar tumor bioluminescence were allocated to 3 groups 4 days after injection. Each group (*n* = 3) was treated 11 times. An in vivo imaging system (IVIS; Xenogen, PerkinElmer, Waltham, MA, USA) was used to assess tumor progression. All of the mice were sacrificed 15 days after the xenograft, and their tumors were excised and processed for H&E staining.

### 2.9. Hematoxylin and Eosin (H&E) and Ki67staining

We used PBS followed by 4% paraformaldehyde (Sigma-Aldrich) for cardiac perfusion in mice. After fixation, brain tissues were embedded in paraffin and sliced at 4–5 μm. Brain tumor sections were deparaffinized with xylene and a graduated alcohol series to water and stained with H&E and Ki67 for microscopic examination.

### 2.10. Global Gene Expression Profile Analysis and Gene Ontology

RNA samples from GBM8401 and LN229 cells were examined using Human OneArray Plus (Phalanx Biotech Group, Hsinchu, Taiwan). The gene expression data are available at the NCBI Gene Expression Omnibus (accession no. GSE174363). GSEA (http://www.broadinstitute.org/gsea/index.jsp (accessed on 1 February 2019) was employed to detect the association between biological processes. The statistical significance was determined based on the normalized enrichment score (NES) and false discovery rate (FDR).

### 2.11. Bioinformatics Investigation of the CGGA Dataset

We performed gene expression and survival analyses with the CGGA dataset using its online tool (http://www.cgga.org.cn/index.jsp (accessed on 1 February 2019) [12]. CGGA is a freely accessible web tool and offers customizable analysis, including association and survival analysis of selected glioma subtypes.

### 2.12. Sphere Formation Assay

Two primary GBM cells, GBM#1 and GBM#2 provided by Ying Kao, were used for the sphere formation assay. A total of 5 × 10^4^ GBM#1 and GBM#2 cells were cultured in 10% FBS DMEM/F12 (Corning, NY, USA) and re-suspended in 2% FBS DMEM medium and an ultra-low 6-well plate (Corning, NY, USA) to allow the formation of spheres. Indicated dosage of diosmin was added to medium one day after plating. We calculated the total number of spheres (>50 μm) under a microscope after 8 days for GBM#1 and 14 days for GBM#2.

### 2.13. Statistical Analysis

Values are expressed as the mean ± standard deviation (SD). Statistical significance of differences between the experimental and control groups was tested using the Student’s *t*-test or one-way ANOVA. Values of *p* < 0.05 were considered significant.

## 3. Results

### 3.1. Diosmin Concentration-Dependently Suppresses GBM Cell Growth and Proliferation

**Figure 1:**
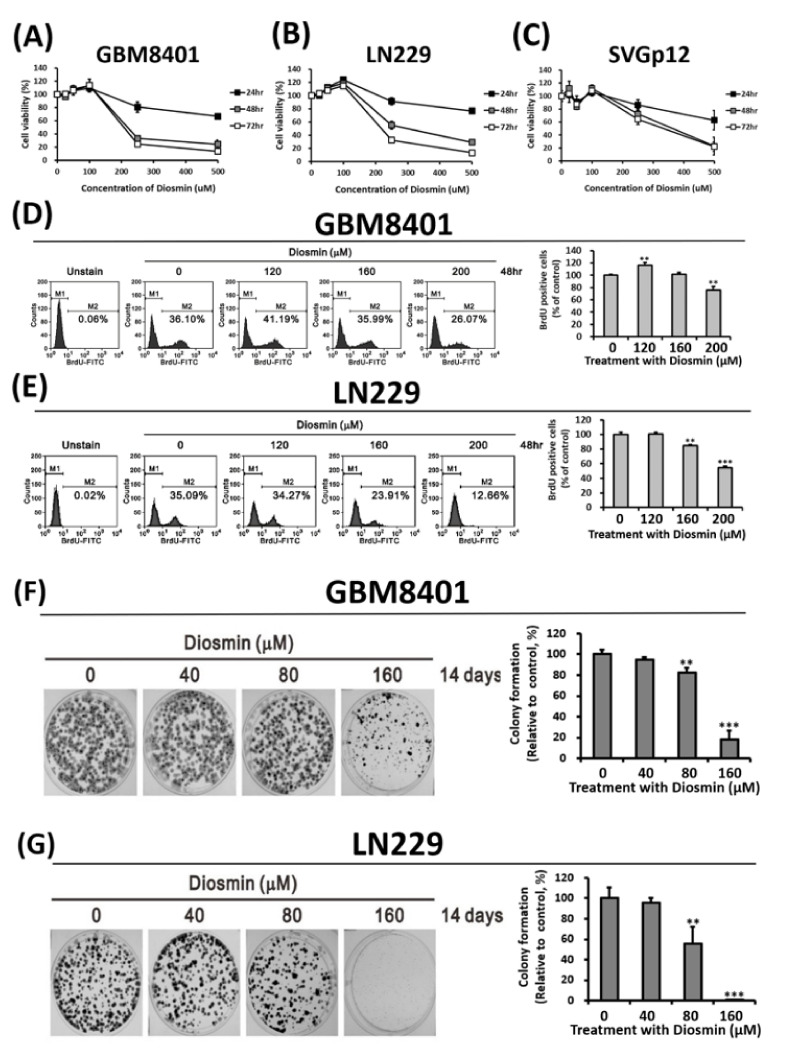
Diosmin concentration-dependently inhibits the growth and proliferation of GBM cells. (**A**–**C**) The viability of GBM8401 (**A**), LN229 (**B**), and SVGp12 (**C**) cells treated with DMSO or different concentrations of diosmin was analyzed with MTS tests after 24 h, 48 h, and 72 h. Data are presented as the mean ± S.D. of at least three independent experiments; ** *p* < 0.01, and *** *p* < 0.001. (**D**,**E**) GBM8401 (**D**) and LN229 (**E**) cells treated with diosmin for 48 h were stained with BrdU and processed for flow cytometric analysis. M1, BrdU-negative cells; M2, BrdU-positive cells. Cells not treated with BrdU were used as a blank. The results are presented as the mean ± S.D. (*n* = 3); ** *p* < 0.01 and *** *p* < 0.001. (**F**,**G**) Clonogenic assay of diosmin-treated GBM8401 (**F**) and LN229 (**G**) cells. The cells were grown in 6-well plates for 14 days, after which they were fixed and stained with crystal violet. Data are presented as the mean ± S.D. of at least four independent experiments; ** *p* < 0.01 and *** *p* < 0.001.

We evaluated the potential anti-GBM effects of diosmin in the GBM8401and LN229 cell lines. The results demonstrated that both GBM cell lines were sensitive to diosmin, as indicated by reductions in cell viability (Figure 1A,B). The diosmin IC50 values in GBM8401and LN229 cells after exposure for 48 h were 218.4 and 299.2 μM, respectively (Figure 1A,B). SVGp12 cells, a normal glial cell line, served as a control and had an IC50 value of 362.6 μM (Figure 1C). These data suggest that GBM cells are more sensitive to diosmin than normal glial cells.

We performed bromo-deoxyuridine (BrdU) incorporation assays to assess the effect of diosmin on GBM cell proliferation. In the presence of diosmin, both GBM8401 and LN229 cells exhibited decreases in BrdU-positive cell fractions, which ranged from 36.10% to 26.07% and from 35.09% to 12.66%, respectively (Figure 1D,E). The suppressive effect of diosmin on cell growth was also validated in clonogenic assays (Figure 1F,G).

### 3.2. Diosmin Induces G1 Phase Arrest Associated with Upregulation of p21 and Downregulation of Cyclin B1, Cyclin D1, CDK1, and CDK2

**Figure 2:**
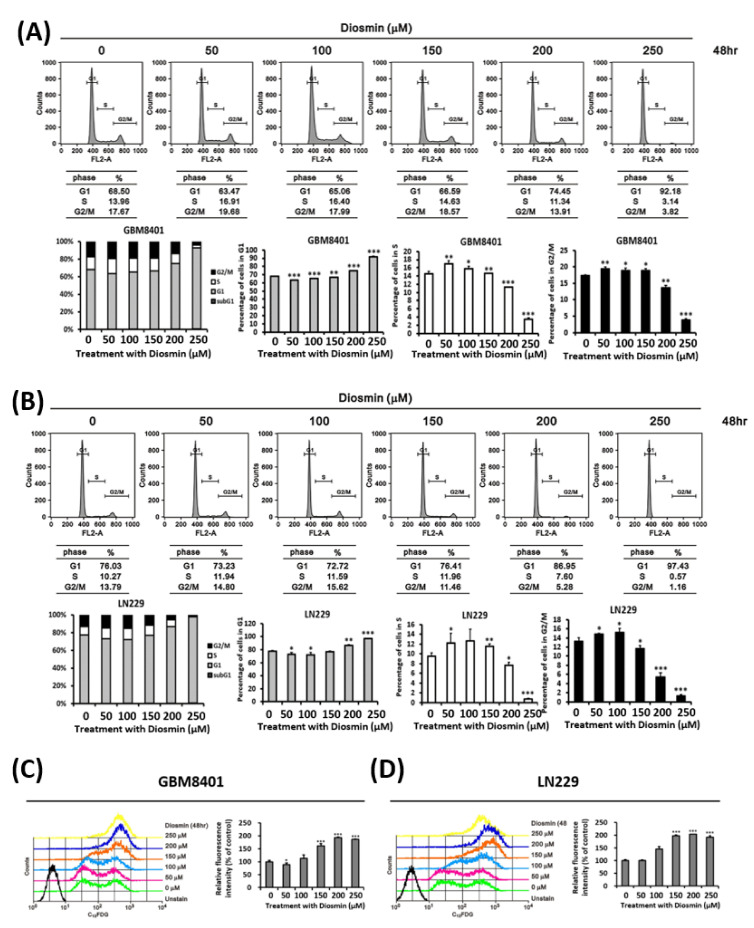
Diosmin induces G1 phase arrest and senescence in GBM cells. (**A**,**B**) Flow cytometric examination of the cell cycle after PI staining of control and diosmin-treated GBM8401 (**A**) and LN229 (**B**) cells. The cell percentages in each phase (subG1, G1, S, G2/M) were estimated with CellQuest software (Becton Dickinson). The results are derived from three independent experiments. * *p* < 0.05, ** *p <* 0.01, and *** *p* < 0.001 vs. controls by using the unpaired, two-tailed Student’s *t*-test. (**C**,**D**) Flow cytometric examination of the senescence after C_12_FDG labeling of control and diosmin-treated GBM8401 (**C**) and LN229 (**D**) cells. The data are shown as the mean ± S.D.; *n* = 3; *** *p* < 0.001 compared to the control group.

**Figure 3:**
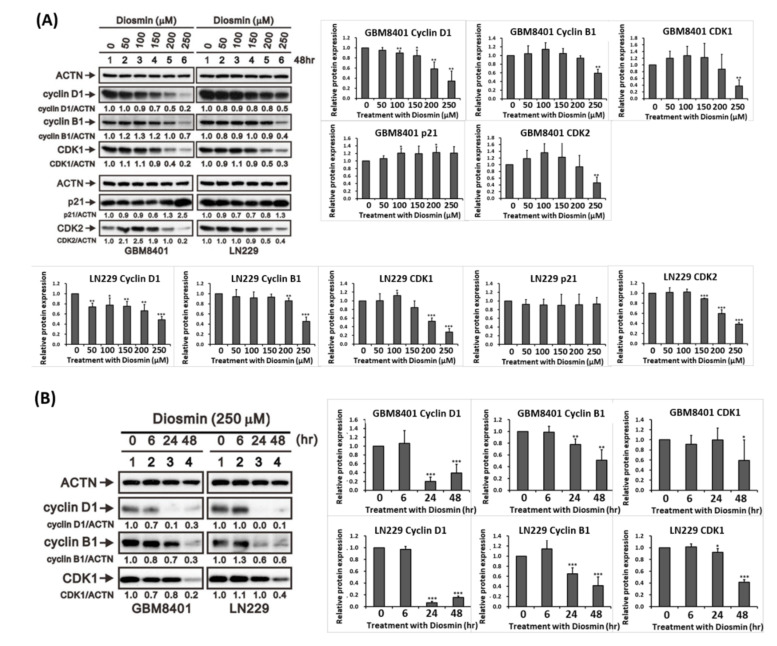
Diosmin induces G1 phase arrest associated with downregulation of cyclin B1, D1, and CDK1. (**A**) Concentration-responsive analysis of cyclin D1, cyclin B1, CDK1, p21, and CDK2 in GBM8401 and LN229 cells (**B**) Time-course analysis of cyclin D1, cyclin B1, and CDK1 in GBM8401 and LN229 cells treated with 250 μM diosmin. The quantification of abovementioned proteins relative to ACTN was shown in the right panel (*n* = 3). * *p* < 0.05, ** *p <* 0.01, and *** *p* < 0.001 vs. control cells.

We next performed cell cycle analysis with propidium iodide (PI)-stained cells to investigate the association between diosmin’s cytotoxic effect and the cell cycle. We found that diosmin significantly and concentration-dependently increased the G1 DNA content in GBM cells, as compared to the untreated control (Figure 2A,B). We further examined senescence that may also be associated with decreased cell proliferation in these diosmin treated glioma cells. There were increased C_12_FDG labeling cells as compared to the untreated control (Figure 2C,D). Subsequent western analyses showed that diosmin elicited upregulation of p21 at high dose and concentration-dependent downregulation of cyclin D1, cyclin B1, CDK1, and CDK2 (Figure 3A). The time-course of the response to 250 μM Diosmin illustrated in the western analysis in Figure 3B shows that expression of cyclin D1 was suppressed after 6 h, while cyclin B1 and CDK1 were suppressed at 24 h and 48 h, respectively. Briefly, diosmin halted the cell cycle at G1 phase by decreasing expression of cyclin D1, cyclin B1, and CDK1.

### 3.3. Diosmin Inhibits Glioma Cells Migration by Decreasing EMT Transcription Factors

**Figure 4:**
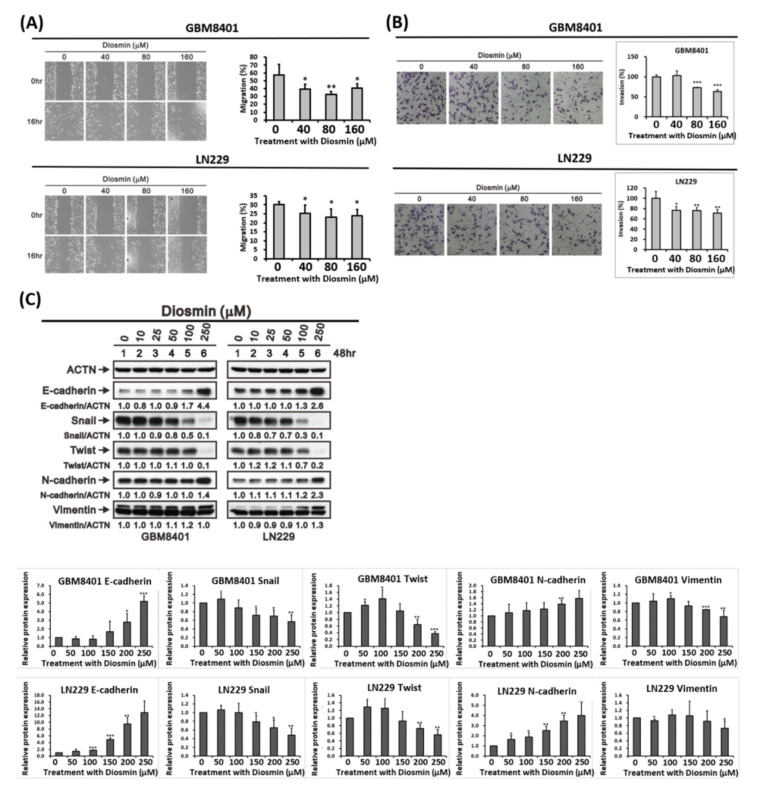
Diosmin reduces the migratory and invasive activities of GBM cells. (**A**) LN229 and GBM8401 cell monolayers in 6 well plates were scratched and then incubated for 16 h with or without the indicated concentrations of diosmin. Wound areas were quantitatively analyzed with ImageJ software and expressed relative to hour 0. (*n* ≥ 3; * *p* < 0.05; ** *p* < 0.01.) (**B**) Invasive activity of LN229 and GBM8401 cells was assessed and quantified with Transwell invasion assays. Invasion areas are presented as the mean ± S.D. (*n* ≥ 3; * *p* < 0.05; ** *p* <0.01, *** *p* < 0.001). (**C**) LN229 and GBM8401 cells were incubated for 48 h with the indicated concentrations of diosmin, after which whole-cell lysates were prepared and subjected to western blot analysis of E-cadherin, Snail, Twist, N-cadherin, and Vimentin expression. The quantification of proteins relative to ACTN was shown in the lower panel (*n* ≥3). * *p* < 0.05, ** *p* < 0.01, and *** *p* < 0.001 vs. control cells.

The phenotypic characteristics of GBM include aggressive invasion and migration. EMT is a reversible biological process in which epithelial cells gain a mesenchymal phenotype characterized by increased cell motility and resistance to genotoxic agents [13]. Glioma cells undergoing EMT acquire the potential to initiate invasion and metastasis [14,15]. For that reason, we tested whether diosmin could alter cell migration and EMT markers in GBM cells. The results of wound healing assays confirmed that diosmin significantly inhibited the migration of LN229 and GBM8401 human GBM cells at the indicated concentrations (Figure 4A,B). Associated with diosmin’s effect on cell migration was the upregulation of the epithelial marker, E-cadherin, and the downregulation of the EMT regulators, Snail and Twist (Figure 4C). These results suggest that diosmin inhibits cell migration by decreasing expression of EMT transcription factors.

### 3.4. Gene Expression Profile Analysis Revealed CCL2 Are Downstream Targets Affected by Diosmin

**Figure 5:**
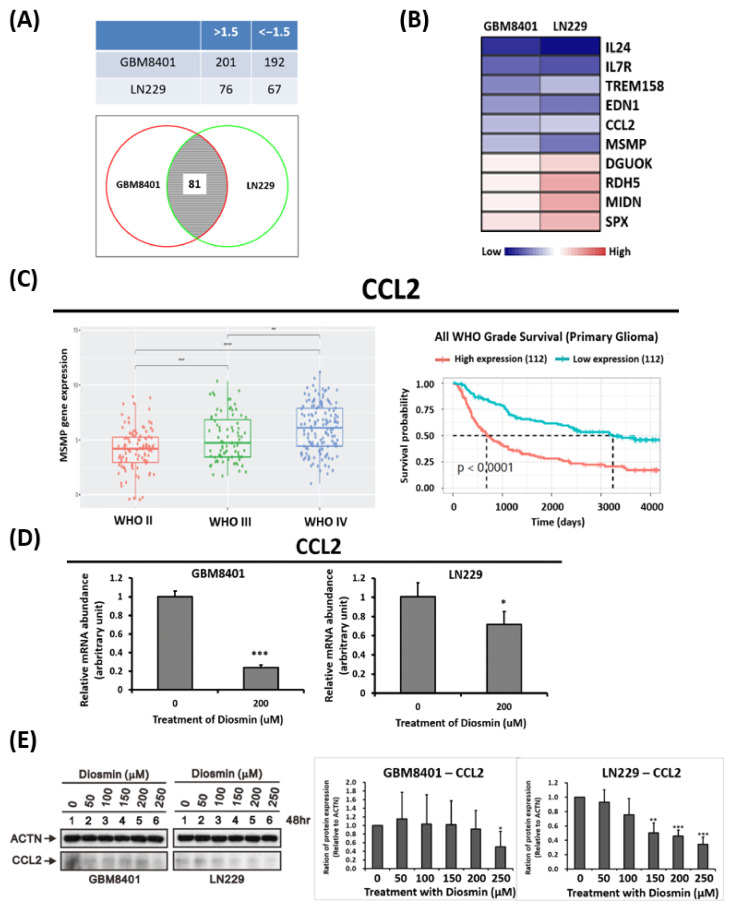
Differentially expressed genes in GBM8401 and LN229 cells treated with 160 μM of diosmin for 48 h. (**A**) Numbers of differentially expressed genes with at least a 1.5-fold change in GBM8401 and LN229 cells are listed (top panel). The Venn diagram shows that expression of 81 genes is influenced by diosmin in both cell types (bottom panel). (**B**) Heat map showing the expression levels of the top 10 mutually upregulated and downregulated mRNAs in GBM8401 and LN229 calls. (**C**) Quantification across WHO grade II-IV (**C**, left panel) and Kaplan–Meier survival analysis (**C**,**D**, right panel) of C-C motif chemokine ligand 2 (CCL2) mRNA levels in the Chinese Glioma Genome Atlas (CGGA) RNA sequencing dataset. ** *p* < 0.01; *** *p* < 0.001; **** *p* < 0.0001 (**D**,**E**) real-time RT-PCR analysis of CCL2 mRNA (**D**) and protein (**E**) expression in GBM8401 and LN229 cells treated with 200 μM of diosmin. The quantification of proteins was shown in the right panel (*n* = 3). WHO, World Health Organization; Bars indicate means ± S.D.; * *p* < 0.05, ** *p* < 0.01, *** *p* < 0.001 vs. control cells.

To understand the molecular mechanisms underlying diosmin’s antitumor effects in GBM cells, microarray was carried out with GBM8401 and LN229 cells treated with and without diosmin. Differentially expressed genes with at least a 1.5-fold change between diosmin-treated and control cells were identified. There were 201 upregulated and 192 downregulated genes in GBM8401 cells and 76 upregulated and 67 downregulated genes in LN229 cells (Figure 5A, top panel). As shown in the Venn diagram in Figure 5A, bottom panel, expression 81 genes were affected by diosmin in both GBM8401 and LN229 cells. Among them, the top 10 diosmin-regulated genes in both cell lines are shown in Figure 5B.

Through a literature review and mining the Chinese Glioma Genome Atlas (CGGA, http://cgga.org.cn/ (accessed on 1 February 2019) [12], we verified that levels of CCL2 are significantly associated with advanced-stage glioma (Figure 5C, left panel). Moreover, high levels of CCL2 predicted poorer survival than low levels of CCL2 (Figure 5C, right panel). Diosmin repressed expression of CCL2 mRNAs and protein in glioma cells (Figure 5D,E) which suggests that this protein is likely downstream to the diosmin target.

**Figure 6:**
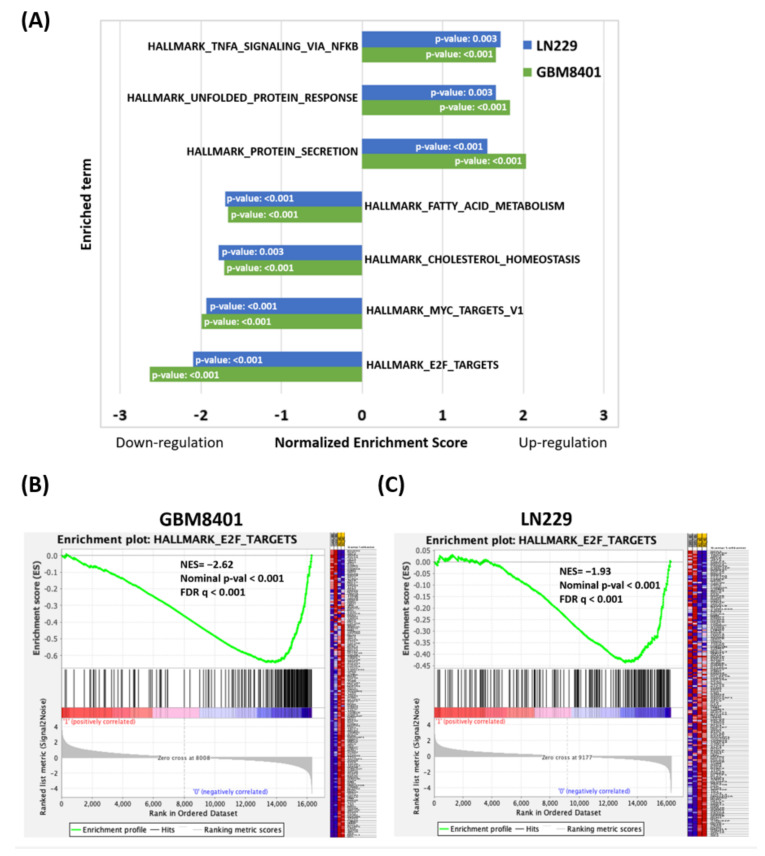
(**A**) Gene set enrichment analysis (GSEA) of the most upregulated and downregulated diosmin-induced phenotypes in GBM8401 and LN229 cells treated with 160 µM of diosmin for 48 h. (**B**,**C**) Enrichment analyses of the most downregulated signal pathway “E2F_targets” in diosmin-treated GBM8401 (**B**) and LN229 (**C**) cells. NES, normalized enrichment score; FDR, false discovery rate.

To identify diosmin-induced, phenotype-specific pathways, we used gene set enrichment analysis (GSEA) to examine diosmin-induced gene expression profiles in GBM8401 and LN229 cells. Pathways with an enrichment score of ≥ 1.5 or ≤1.5 were recruited. As shown in Figure 6A, the most enriched upregulated gene sets were related to TNFA signaling via NFKB, an unfolded protein response, and protein secretion. The most enriched downregulated gene sets were linked to E2F targets, MYC targets, cholesterol homeostasis, and fatty acid metabolism. The E2F target pathways are the most changeable of all signaling pathways. GSEA showed that most genes contributing to E2F target signaling were downregulated (Figure 6B,C).

### 3.5. Diosmin Is a Potent Inhibitor of Autophagic Flux 

**Figure 7:**
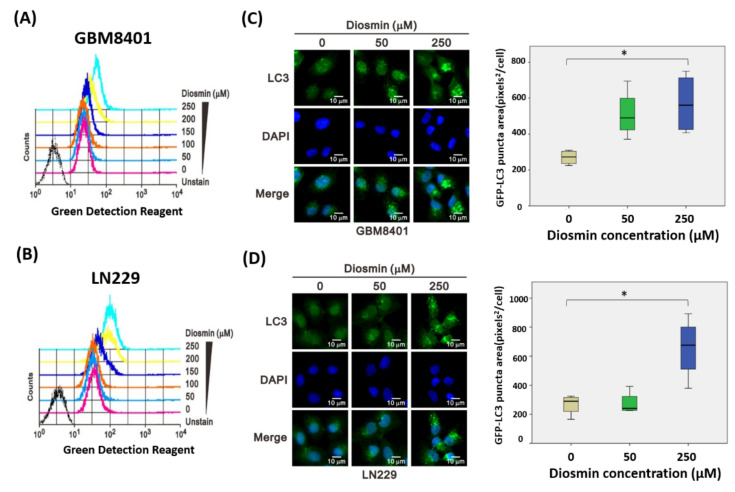
Diosmin induces LC3-II expression in GBM cells. (**A**,**B**) Flow cytometric analysis of the number of autophagic vacuoles in GBM8401 and LN229 cells exposed to increasing concentrations of diosmin for 48 h. (**C**,**D**) Left: LC3-II immunofluorescence imaged after incubation of GBM8401 (**C**) and LN229 (**D**) cells for 48 h with the indicated concentrations of diosmin. Representative images used for thresholding quantification are shown. Right: Box plots showing the total areas of the green fluorescent puncta within the cells (mean ± SEM of 4 independent experiments; * *p* < 0.05, Student’s *t*-test).

Because cell cycle assays revealed no obvious change in the proportion of sub-G1 cells (Figure 2), we hypothesized that diosmin inhibits cell growth through a different mechanism, i.e., autophagy. To determine whether diosmin influences autophagy, we used an autophagy detection kit to assess autophagic activity flow cytometrically. As shown in Figure 7A,B, diosmin concentration-dependently increased the fluorescent signal from both GBM cell lines. Furthermore, confocal microscopic assessment of immunostained LC3B revealed enhancement of the LC3B signal and the presence of classic punctate structures in both LN229 and GBM8401 cells after exposure to 50 and 250 μM diosmin for 48 h (Figure 7C,D).

**Figure 8:**
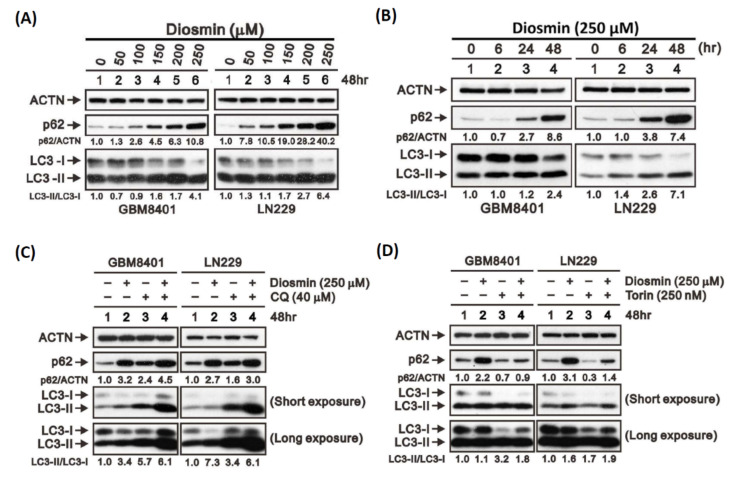
Diosmin is an autophagy inhibitor in GBM cells. (**A**) Western blotting of p62, LC3-I, and LC3-II in lysates from GBM8401 and LN229 cells incubated for 48 h with the indicated concentrations of diosmin. (**B**) Western blotting of p62, LC3-I, and LC3-II in lysates from GBM8401 and LN229 cells indicated with 250 μM of diosmin for the indicated times. (**C**,**D**) Western blotting of p62, LC3-I, LC3-II, and β-actin in lysates from GBM8401 and LN229 cells incubated for 48 h with 10 μM chloroquine (CQ) and/or 250 μM diosmin (**C**) or 100 μM torin and/or 250 μM diosmin (**D**).

Initiation or inhibition of autophagy can cause elevation of LC3-II, but the induction of autophagy is most associated with a decrease in p62 [16]. Western blotting showed that levels of LC3-II were significantly and concentration-dependently increased in LN229 and GBM8401 cells after treatment with diosmin for 48 h (Figure 8A) and that 250 μM of diosmin elicited a time-dependent increase in LC3-II levels (Figure 8B). These changes in LC3-II levels indicate that diosmin affected autophagy in these GBM cells. However, diosmin exposure also led to concentration- and time-dependent increases in p62, not its degradation (Figure 8A,B). This suggests that diosmin inhibited an autophagic reflux.

To further validate diosmin’s function as an autophagic flux blocker, we challenged the GBM cells using torin 1, an autophagy inducer, and CQ, an autophagy blocker. Like diosmin, CQ induced expression of both p62 and LC3-II, and the combination of diosmin and CQ had an additive effect on p62 and LC3-II expression (Figure 8C). By contrast, torin-1 induced p62 degradation and LC3-II accumulation (Figure 8D), and diosmin partially inhibited torin 1-induced p62 degradation (Figure 8D). These results confirm that diosmin acts as an inhibitor of autophagic flux.

### 3.6. Diosmin Diminishes Tumor Bulk in a Xenograft Mouse Model and Tumor Sphere Formation in Clinical Specimens

**Figure 9:**
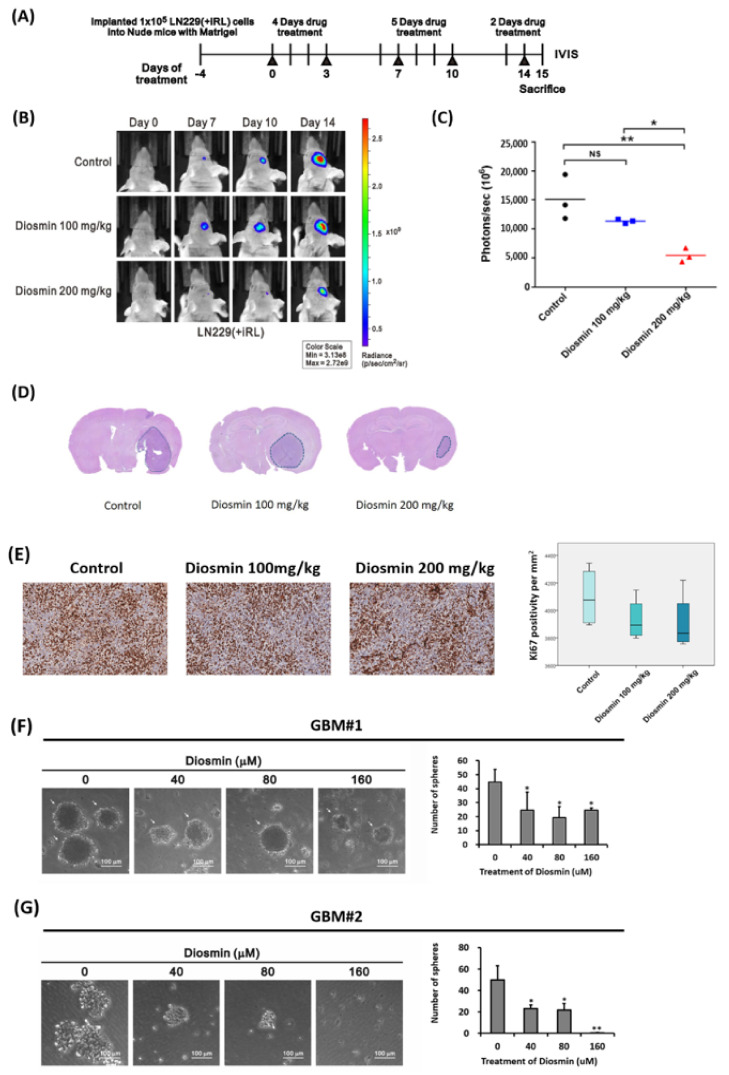
Diosmin inhibits LN229 growth in a mouse orthotopic xenograft model and tumor sphere formation in clinical specimens. (**A**) Experiment timeline for the mouse LN229-iRL orthotopic xenograft model. (**B**) Progression of tumor development estimated from bioluminescence captured with an in vivo imaging system (IVIS). (**C**) Quantitative analyses of in vivo bioluminescent imaging data from the three groups. *n* = 3 for each group; ns, not significant; * *p* < 0.05; ** *p* < 0.01 (**D**,**E**) Representative images of H&S (**D**) and Ki67 (**E**) stained brains from representative mice in the three groups. The sections were prepared 15d after intracerebral injection of LN229-iRL cells. Right: Box plots showing the Ki67 positivity per mm^2^ (mean ± SEM, *n* = 3). (**F**,**G**) Representative photographs of tumor spheres formed from 2 fresh clinical GBM specimens. Scale bar 200 μm. The cells treated with indicated concentrations of diosmin were cultured in 6-well plates for 8 days for GBM#1 and 14 days for GBM#2. Data are presented as the mean ± s.d. of at least four independent experiments; * *p* < 0.05, and ** *p* < 0.01.

To further assess the anticancer effects of diosmin, we orthotopically transplanted LN229 (+iRL) glioma cells into the brains of nude mice. The mice were assigned to three groups—control, low-dose diosmin (100 mg/kg), and high-dose diosmin (200 mg/kg)—4 days after grafting LN229 (+iRL) cells (day 0). Diosmin was administered intraperitoneally 11 times from day 0 to day 15 (Figure 9A), after which brain tumor development was evaluated using an in vivo imaging system (IVIS), H&S and Ki67 staining. Both IVIS and H&S staining showed that administration of high-dose diosmin (200 mg/kg) led to significant reductions in tumor size (Figure 9B–D). Furthermore, there is a trend toward decreased Ki67 staining in tumors from diosmin treated group (Figure 9E).

To advanced validate the anti-GBM effect of diosmin on tumor spheres, we used GBM#1 and GBM#2 derived from two fresh clinical specimens for sphere formation assay. Representative images of primary GBM spheres after treated with diosmin were shown in Figure 9F,G. Diosmin significantly inhibited the number of tumor spheres (Figure 9F,G, right panel). Based on these results, diosmin could suppress the tumorigenicity of GBM cells.

## 4. Discussion

**Figure 10:**
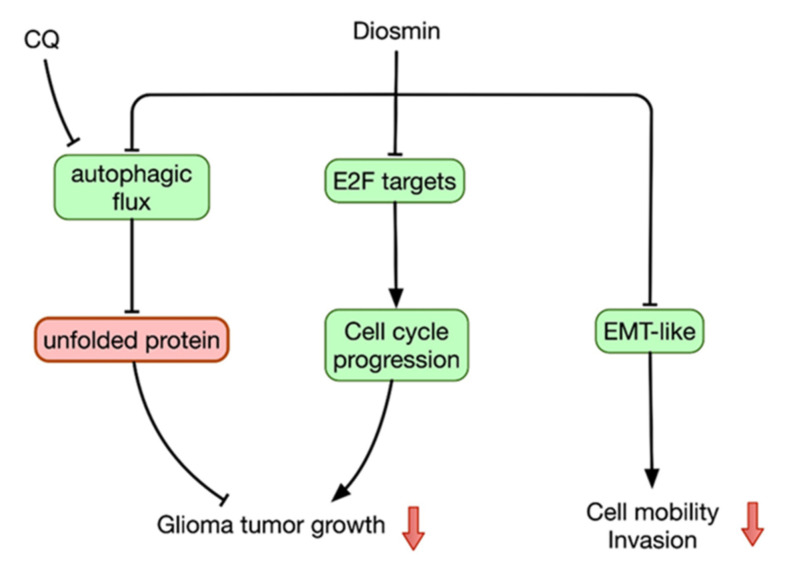
Proposed model for anti-glioma mechanism of diosmin. EMT-like, epithelial-mesenchymal like transition.

Diosmin is one of the flavonoids isolated from citrus, and has been applied for the treatment of stasis dermatitis and venous ulcers caused by chronic venous insufficiency [17] as well as for hemorrhoids. [18]. Moreover, diosmin may also possess anticancer activity [8]. This is the first study to investigate diosmin’s antitumor effects in GBM through in vitro and in vivo experiments. Our data show that diosmin concentration-dependently inhibits GBM cell growth through induction of cell cycle arrest at G1 phase and inhibition of autophagic flux. Diosmin also impedes migration and invasion by GBM cells through suppression of EMT-like process. Thus, diosmin diminished GBM development by hindering both GBM cell proliferation and migration/invasion (Figure 10).

Migration and invasion are critical events in GBM progression and are also associated with recurrence. In particular, enhanced activity in the EMT pathway, which includes glial-mesenchymal transition, correlates with poor survival due to aggressive invasion and poor responses to chemoradiotherapy [19,20,21]. Chen et al. reported that the low levels of EMT-related gene markers in GBM tumor samples are correlated with increased time span between initial treatment and recurrence [19]. Rao et al. reported that enhanced expression of genes engaged in the EMT pathway is the most significant feature of GBM as compared to Grade III anaplastic astrocytoma [22], which suggests that EMT is a key factor influencing prognosis in GBM patients. We observed that the reduction in GBM cell migration and invasion elicited by diosmin was associated with increased expression of the epithelial marker, E-cadherin, and decreased expression of the EMT transcription factors, such as Snail and Twist. Moreover, not only are Snail and Twist transcriptional factors involved in inducing EMT, they are also involved in augmenting GBM stemness, leading to cancer progression and therapeutic refractoriness [21,23].

Autophagy is a multistage process controlled by various autophagy-associated proteins. In the present study, flow cytometry, immunohistochemistry, and western blotting experiments showed that diosmin upregulated expression of LC3-II in GBM. However, it also increased the expression of p62, which suggests that diosmin likely inhibits autophagic flux in human GBM cells. Consistent with that idea, the autophagic blocker CQ mimicked the effects of diosmin, in turn increasing the levels of both p62 and LC3-II. Furthermore, the combination of diosmin and CQ had an additive effect on p62 and LC3-II levels. On the other hand, the autophagic inducer torin-1 also increased LC3-II accumulation but decreased levels of p62. At present, CQ is the most well-known autophagic inhibitor that also exerts antitumor effects on glioma cells, including diminishing invasiveness in GBM cells [24] and dose-dependent inhibition of cell growth [25]. Furthermore, one double-blind clinical trial reported GBM patients receiving CQ plus standard treatment, which consisted of surgery and chemoradiotherapy, and had better mid-term survival than the placebo group [26]. In the present study, we found that the antitumor effects of diosmin on GBM cells are similar to those of CQ, and we confirmed the antitumor effect of diosmin in vivo using a mouse xenograft model. Notably, Buccarelli et al. reported that suppressing autophagy increased the vulnerability of GBM stem cells to temozolomide (TMZ) [27]. This suggests that future testing of the combination of diosmin with TMZ and radiation for treatment of GBM is warranted.

In this study, we found that the E2F targets pathway is the most changeable and downregulated in GBM cells treated with diosmin. E2F family members play critical roles during G1/S transition in the cell cycle [28]. Consequently, repression of the E2F pathway may partially explain the diosmin-induced G1 cell cycle arrest observed in this study. In addition, the E2F pathway has been connected to the regulation of autophagic activity [29]. Jiang et al. reported that suppression of E2F1 by RB1 in cells contributes to increased levels of autophagy [30]. However, Polager et al. reported the E2F1 promotes autophagic activity by upregulating several autophagic factors, including ATG1, ATG5, and LC3 [31]. This suggests that the precise role of the E2F pathway in autophagic regulation may depend, in part, on environmental factors. Further studies investigating the changes in E2F family members caused by diosmin in GBM cells will be needed to clarify this issue. The significant upregulation of unfolded protein responses (UPR) pathway is also noted in diosmin-treated GBM cells. UPR plays as a protective system against unfolded or misfolded proteins and is coordinated by endoplasmic reticulum membrane sensors [32]. Like autophagy, the UPR pathway is a double-edged sword in antitumor therapy. UPR is initially elicited under stress state and can cause either survival or death reliant on trigger and duration [33]. Dastghaib et al. reported that cotreatment with simvastatin and temozolomide could induce U87 and U251 glioma cell death by inducing UPR and inhibited autophagy flux [34]. Moreover, simvastatin–temozolomide-induced inhibition of autophagic flux in GBM cells is associated with IRE1 and PERK signaling arms of the UPR [34]. This report provides potential signaling arms of UPR for explanation of autophagic flux inhibition in diosmin-treated glioma cells. This will be worth for further investigation in the future.

We used gene microarray analysis and discovered that CCL2 is the potential downstream target of diosmin. CCL2 is one of the CC chemokine group and has monocyte chemoattractant ability to promote inflammatory responses [35]. CCL2 overexpression is observed in several types of cancer, including glioma and prostate cancer. Lu et al. reported increased CCL2 expression in glioma tissue compared with healthy tissue. Moreover, CCL2 silencing by siRNA could repress the growth and angiogenesis in the U251 glioma cell line [36]. Shono et al. also reported that the anti-glioma effects of celecoxib are associated with CCL2 knockdown [37]. In prostate cancer studies, CCL2 overexpresses in the tumor microenvironment and acts as an important role in tumorigenesis and invasion [35]. Moreover, Roca et al. found that CCL2 protects prostate cancer PC3 cells from autophagic death by upregulation of survivin [38]. This report provides a potential molecular mechanism between diosmin and autophagic control. However, more studies are required to validate this issue.

Soares et al. also reported that diosmin significantly inhibited the viability of three different GBM cells, except healthy human astrocytes. This result provided another evidence for diosmin’s anti-GBM ability. They found that diosmin provoked caspase-dependent apoptosis in GBM cells, as evidenced by the TUNEL assay [39]. However, we did not observe significantly increased apoptosis in this study. The difference of tested cell lines may partially explain this dissimilar result.

## 5. Conclusions

Our data demonstrate the anticancer impact of diosmin on the GBM8401 and LN229 human GBM cell lines. These effects reflect diosmin’s ability to inhibit cell cycling, EMT, and autophagic flux. Diosmin exerts these effects without serious toxicity toward the control SVG-p12 astrocyte line, though higher concentrations did diminish astrocyte viability. This study provides potentially helpful support for the development of new therapies for the treatment of glioma. Future experiments will be required to gain further insight into diosmin’s antitumor effects on TMZ- and radiation-resistant GBM cells.

## Data Availability

The data presented in this study are available on request from the corresponding author.
